# Dynamic expressions of monocyte chemo attractant protein-1 and CC chamomile receptor 2 after balloon injury and their effects in intimal proliferation

**DOI:** 10.1186/s12938-015-0030-8

**Published:** 2015-06-11

**Authors:** Zhigang Huang, Yuebing Li, Lili Niu, Yang Xiao, Xiaodong Pu, Hairong Zheng, Ming Qian

**Affiliations:** Emergency Department, Peking University Shenzhen Hospital, Shenzhen, 518036 China; The Second Affiliated Hospital of Zhejiang Chinese Medical University, Hangzhou, 310005 China; Paul C. Lauterbur Research Center for Biomedical Imaging, Institute of Biomedical and Health Engineering, Shenzhen Institutes of Advanced Technology, Shenzhen, 518055 China

**Keywords:** Balloon injury, Intima proliferation, Monocyte chemoattractant protein-1, CC chemokine receptor 2

## Abstract

**Objective:**

The dynamic expressions of monocyte chemo attractant protein-1 (MCP-1) and CC chamomile receptor 2 (CCR2) after balloon injury and their effects in intimal proliferation were discussed. In this study, the expression of MCP-1 and its receptor during the intimal proliferation in rat artery after balloon injury were studied.

**Methods:**

Using the model of balloon injury of rats’ arteries, the changes of intimal proliferation were observed with optical microscopy and the expressions of MCP-1 and CCR2 at different times were examined with the methods of RT-PCR and immunohistochemistry. The expressions of MCP-1 and CCR2 in the arterial tissues were detected using reverse transcription polymerase chain reaction (RT-PCR) and analyzed by semi-quantitative method.

**Results:**

The expressions of MCP-1 and CCR2 mRNA began to gradually increase after balloon injury. The MCP-1 reached to the peak on the first day, but decreased gradually later on. Expressions of CCR2 mRNA began to increase on the first day and reached to the peak on the 7th day, but then started to decrease gradually until 28th day when we can still detect it. The expressions of MCP-1 proteins began to increase gradually after balloon injury and were obviously detected in the VSMC on the 4th and 7th day, until 14th day when we can still detect it clearly in the proliferating intima.

**Conclusion:**

The dynamic expressions of MCP-1, MCP-1 proteins and CCR2 mRNA after balloon injury were shown to play an important role in intimal proliferation.

## Introduction

Ever since percutaneous transluminal coronary angioplasty (PTCA) was performed by the Swiss physician, Gruentzig [1] in 1977, it has been proven to have many advantages such as resulting in small trauma, ease of implementation, ability to relieve symptoms, and is rapid and reliable [2,3]. As such, it rapidly popularize and partially replace coronary artery bypass graft (CABG), and now it has become one of the more simple and effective methods, which are used to treat cardiovascular disease. But many clinical researchers find that acute coronary occlusion and restenosis easily occur after PTCA and the rate of restenosis is as high as 30% to 50%, which affect its long-term effect and clinical application [4]. From 1987 till now, stent placement, which is regarded as a new technology, is used in clinic all the time. Based on the supporting role of stents, it can significantly prevent and treat vascular remodeling which is caused by vascular elastic recoil and can effectively reduce the incidence of restenosis after PTCA [5].

Restenosis refers to a repair response that occurs after local vascular injury. Restenosis is reconstruction and remodeling of partial vascular that is mediated by a variety of cytokines and growth factors; it is caused by the migration, proliferation and apoptosis of vascular smooth muscle cells (VSMC) and secretion and accumulation of extracellular matrix (ECM). It is also responsible for the changes of structures and functions of vascular wall, which are caused by abnormal expressions of a series of genes [6]. The gold standards of diagnosis of restenosis are coronary angiography and ultrasound inspection in coronary arteries. When loss of diameter of lumen is more than 50% after coronary dilatation, it related to restenosis occurrence in vessels [7,8].

In simple angioplasty of balloon expansion, the mechanism of restenosis mainly may be vascular elastic recoil in early stage, proliferation of VSMC and secretion of ECM in metaphase and tardive and contractile vascular remodeling, which is caused by repair of damaged vessels in advanced stage. But the occurrence of restenosis after stent placement is mainly related with proliferation of VSMC and secretion of ECM.

There are injuries and exfoliation of endotheliums in each PTCA. Injuries of endothelium are the initial factors of the occurrence of restenosis [9,10]. In recent years, large numbers of studies have proved that inflammatory response is a key part of the occurrences and developments of many cardiovascular diseases. Many cytokines take part in inflammatory response and constitute complex networks of cytokines. Some inflammatory markers may be predictive factors of cardiovascular diseases, among the markers, MCP-1 plays a significant role in inflammatory cells, especially in the chemoattractant and activation of monocytes in inflammatory responses of cardiovascular diseases [11-13].

In this experiment, we used model of balloon injury of rats to observe expressions of monocyte chemoattractant protein-1 (MCP-1), CC chemokine receptor 2 (CCR2) and MCP-1 proteins at different times after balloon injury. The purpose of the experiment is investigating their possible roles in intimal hyperplasia.

## Materials and methods

### Instruments and reagents

A number of instruments and reagents were used, including Super-clean Bench (SuJing VS-1300 V), Gel Imaging Instrument (PHARMACIA UDS-CL), Analytic Apparatus of Protein and Nucleic Acid (BECKMAN DU640), PCR Instrument (Eppendorf-personal, Gradient), Low-temperature and High-speed Centrifuge (Heraeus-Biofuge), Balloon Catheter (Cordis 2.0 × 15 mm), Pressure Pump (Cordis), Electrophoresis Apparatus, Cryopreservation Refrigerator (from MDF392 Sanyang Company), Microscope (Olympus), Ultraviolet Spectrophotometer (Shanghai Analysis Instrument Factory Type 751-GW), TRIZOL (Invitrogen), Reverse Transcription Kit (Promega A3500), PCR Reagents (Takara Company), Immune Tissue Staining Kit, and Elastic Fiber Staining Kit.

### Animal specimens

Forty-eight rats were used in this study. The rats were provided by Shanghai Animal Center Laboratory of China Medical Sciences Academy. The rats’ weights ranged from 350 to 450 grams, with an average age of 12 to 14 weeks. These rats were divided randomly into six groups, and formed one sham group and five balloon injury groups. The sham group consists of 8 rats and the balloon injury group was evenly divided into 5 subgroups, which were respectively named as 1st day group, 4th day group, 7th day group, 14th day group, 28th day group.

### Production of animal model and drawing materials

Rats were raised and used in the Animal Laboratory of Peking University Shenzhen Hospital. Two rats were placed into each cage which were considered as one group, then put all cages into laminar flow rack which was placed in the room whose temperature was 21 to 23°C. Rats were fed normal diets for 7 days at a dose of 20 grams per day and fed with water freely. Before operation, rats were restricted from eating and drinking for 24 hours. Subsequently, 2% Sodium Pentobarbital whose dose was 2 ml/kg (0.04 g/kg) was injected into their abdominal cavity to anesthetize the rats.

The rat’s limbs and heads were fixed. Its fur was sheared off at the middle of the neck and was conventionally disinfected using a spread towel. An incision with length of about 2.5 cm were cut in the middle of the neck above the sternum about 1 cm. The subcutaneous tissues were separated, and the sternocleidomastoid was removed. The common carotid sheath was opened to separate the left common carotid artery (CCA). Later on, the distal end of CCA was ligated with silks, and pulled up in the proximal end with silks to prevent blood flow. Also a few drops of 2% Lidocaine Hydrochloride were dripped on the common carotid artery to dilate vessels. A small incision was cut in the CCA and the guide wires and balloon (2.0 mm × 15 mm) were inserted into the distal end of abdominal aorta from the proximal end of the left CCA. The balloon was filled at 8 atmospheric pressures and rotated for 120 degrees, in order to peel artery endothelial. This procedure was repeated for three times. Then, the balloon was pumped to negative pressure and removed the guide wire with balloon out of the abdominal aorta. In the end, the rat was put into a cage living freely. The operators executed the rats respectively on the operating day, 1st, 4th, 7th, 14th and 28th days for experiment. Treatment measures of sham group were identical to balloon injury group without inserting the guide wire and balloon. When the phenomenon of denudation and hyperplasia of intima appeared in histopathological examination, it indicated that the model was developed.

### Indicators and measurements

Formalin with concentration of 10% was used to fix the abdominal aorta that was above 2 to 3 cm from iliac artery bifurcation in the rats. The specimen was embedded with paraffin, and routine pathological serially section was performed. The slice was 4 μm thick. After that, H&E staining and Elastic Fiber staining were performed. Finally, a vascular morphological detection on the section was made.

The procedure for the morphological observation and image analysis of vascular hyperplastic intima was as following. The condition of intimal hyperplasia was observed with microscopy. The images within sections and in the images vascular cross-sectional was recorded. Then the images were processed by an Computer Image Analysis System and the intimal thickness, media thickness were measured and the ratio of intimal thickness and media thickness was calculated.

The remaining aorta was put into a liquid nitrogen pot immediately, and was stored in ultralow refrigerator at a temperature below minus 80°C. The stored aorta was used for detecting the expressions of genes (MCP-1, CCR2mRNA) by RT-PCR. The mRNA sequences of target genes MCP-1 and CCR2 (Serial number respectively is M57441and NM021866) and internal gene GAPDH were obtained from Genbank, Subsequently upstream and downstream primers of target genes were designed by Primer Express and specific analysis was made on each primer using BLAST engine to ensure that each primer had no homology to other known genes in the gene pool of rats. Primer sequences were respectively showed in below. Upstream of MCP-1 (288 bp) was 5′—CAATGAGTCGGCTGGAGAAC —3′ and downstream of MCP-1 (288 bp) was 5′—CAGAAGTGCTTGAGGTGGTTG—3′, upstream of CCR2 (236 bp) was 5′—GAGGCATAGGGCTGTGAGG—3′ and downstream of CCR2 (236 bp) was 5′—GATACCTTCGGAACTTCTCACC—3′, upstream of GAPDH (143 bp) was 5′—GGCTGAGAATGGGAAGCTGGTCAT—3′ and downstream of GAPDH (143 bp) was 5′—CAGCCTTCTCCATGGTGGTGAAGA—3′. The design of primer was completed by Medical Molecular Biology Center of Fujian Medical University, and the synthesis of primers was performed by the Shanghai Boya Corporation.

The total RNA was extracted referring to the process of instructions of extraction of RNA kit that was provided by Gibco Company, and the steps were as below. First, the frozen aorta was taken from ultra-low refrigerator, weighed, and rinsed with sterilizing water. Then it was cut into pieces and transferred into glass tubes. Subsequently Trizol was added at a dose of 100 mg per ml and speeded the tubes. After that, the tubes were put into ice bath for 10 to 15 minutes. Second, the slices were put into a new EP tube whose volume was 1.5 ml, and was then spun at 3000 g at 4 centigrade for 10 minutes. The supernatant fluid was removed to another EP tube and placed at indoor temperature for 10 to 15 minutes. After that, Chloroform was added at a dose of 0.2 ml per ml of Trizol. The tube was shook for 15 s and placed the tube for 5 minutes at room temperature. Later on it was spun at 12000 g at 4 centigrade for 15 minutes. The upper aqueous phase was carefully drawn and put into a new EP tube. Third, Isopropanol was added into the new tube and the tube was rolled slowly and then placed at room temperature for 10 minutes. It was spun again at 12000 g at 4 centigrade for 10 minutes. Then, white precipitate can be seen on the bottom of EP tube. Fourth, the supernatant liquid was discarded and 1 ml 75% Ethanol was added. The tube was shook to wash precipitate adequately, and was subsequently spun at 11000 g at 4 centigrade for 5 minutes. Fifth, the Ethanol was absorbed and dried the tube for 10 minutes with air. DEPC water was added into the tube. Sixth, the RNA was dissolved in water whose volume was 50 μl and whose nuclease was removed. Next it was placed in minus 20°C to cryopreservation and standby. Seventh, the total extracted RNA was taken to estimate the quantitative and purity of the RNA using ultraviolet spectrophotometer and the value of OD260 and OD280 were measured. Eighth, the ratio value of OD260 and OD280 were measured. If the values of all specimens were 1.7 to 2.0, the RNA purity was considered to be good. Finally, electrophoresis was made with 1% Agarose Gel on total extracted RNA. Subsequently two clear bands of rRNA could be observed which respectively were 28S and 18S, suggesting that extracted RNA was not degraded.

The semi-quantitative reverse transcription - polymerase chain reaction (RT-PCR) was performed referring to the instructions of reverse transcription kit and PCR reagents which were provided by Promega and Takara Corporation. First, we compound the first chain of cDNA. The reaction systems had total RNA of 2 μg, 10 × buffer of 2 μl, 25 mmol/L MgCl2 of 4 μl, 10 mmol/L dNTPs of 2 μl, random primers of 100 ng, Rnasin (RNA enzyme inhibitor) of 20 U, AMV reverse transcriptase of 15 U and ddH2O of 20 μl. The reaction conditions were 70°C for 10 minutes, amplification at 42°C for 60 minutes, inactivating AMV at 95°C for 5 minutes. Products of cDNA were placed in subzero 20°C. Secondly, we performed PCR amplification. The reaction systems had cDNA of 3 μl, 10 × PCR Buffer of 2.5 μl, 25 mmol / L MgCl2 of 1.5 μl, 10 mmol/L dNTP of 0.5 μl, EXTaq enzyme of 1.5 U and deionized water of 25 μl. The reaction conditions were predenaturation at 4°C for 5 minutes, denaturation at 94°C for 30 s, annealing at 60°C for 30 s and extension at 72°Cfor 60 s, total cycles of 30 and further extension at 72°C for 5 minutes.

Finally, the electrophoresis and image scanning were implemented and the steps were assessed as follows. First, we took PCR products of 10 μl to make electrophoresis with 2% Agarose Gel whose voltage dropped 5 V per cm. Here, the process lasted for 60 minutes. Second, we stained them with Ethidium Bromide. Third, we scanned the products by the Image Master VDS-CL of Gel Imaging System. At the fourth step, we calculated gray integral of electrophoresis bands of PCR products and did standard correction by using gray integral of GAPDH. Relative quantities of PCR products of target genes was the ratio value of gray integral of electrophoresis bands of target genes and gray integral of GAPDH.

The immunohistochemical detection was performed and semi-quantitative analysis of immunohistochemical results. Referring to the instructions of UltraSensitiveTM SP hypersensitivity kit (Kit-9701) of Fujian Maixin Biotechnology Development Limited Company, we uniformly sliced 6 to 10 slices on the already embedded vessels and the thickness of each slice was 4 μm. After that, we attached slices to object slides which had already been treated by Poly-lysine and grilled slides for 4 hours at 60°C to standby. First, paraffin sections were took off the wax by Xylene for 15 minutes for twice. Later on, we used Gradient Alcohol (100% alcohol for 3 minutes for twice, 95 % alcohol for 3 minutes, 85% alcohol for 3 minutes) to hydrate them.. Second, we used Phosphate Buffered Saline (PBS) whose PH was 7.4 to wash them 3 times, 3 minutes each time. Third, we exposed antigens with high temperature and high pressure of tissue antigen retrieval method: we put a certain amount of citrate buffers whose PH was 6.0 into a pressure cooker, next used fire heat it to boiling, at this time, we placed tissue sections into plastic sliced rack which resists high temperature, later on put the rack into already boiling buffer and covered and fastened the pressure valve, continue heated to jet and lasted this condition for 1.5 minutes, we removed the pressure cooker from heat, let it natural cool to room temperature, finally removed the valve and opened the pressure cooker and took out the object slides. Fourth, we washed the slides with distilled water, then rinsed them for three times with PBS and 3 minutes each time. Fifth, drying the moisture surrounding tissues, we dripped 3% Hydrogen Peroxide of 50 μl to each organization and incubated for 10 minutes at room temperature to block activity of endogenous peroxidase, after that, rinsed three times with PBS, 3 minutes each time. Sixth, we prepared and dripped Rabbit Anti-mouse Antibody (Boster, Rabbit Anti-MCP-1, product number is BA1255) based on a usage of 20 ul whose concentration was 1:50 to each organization and incubated it for 60 minutes at room temperature. After that we rinsed them three times with PBS, 3 minutes each time. Seventh, drying moisture surrounding tissues, we dropwise dripped ready-to-use reagents MaxVision of 50 μl to each organization and incubated for 10 to 15 minutes at room temperature. When this was done, we rinsed them three times with PBS and for 3 minutes each time. Eighth, drying moisture surrounding tissues, we used fresh DAB solution, and following that, observed color development for about 5 minutes by microscopy. Then, we washed the items using tap water and dyed them using Hematoxylin. Finally, we rinsed them using PBS. After all these steps, we dehydrated them using Graded Alcohol, cleared them through Xylene and fixed them via neutral gum. The positive reaction gives a brown cytoplasm.

The semi-quantitative analysis of immunohistochemical results was stated. In the Color Image Analysis System, we randomly selected five high-power fields for each slice and measured the areas and mean grays of positive signals within a certain area for each field, next, calculated the index of positive signals of antigen using average value for each slice. The formula is the multiplication of the index of positive signals of antigen is equal to areas of positive signals with the mean grays of positive signals, and then division by the measured areas and followed by a multiplication of 100. Results of semi-quantitative examination of expressions of MCP-1 proteins are replaced by indices of positive signals.

### Ethical approval

All animal experiments were approved by the committees for animal experiments of Shenzhen Institutes of Advanced Technology and conducted according to the 2 institutional ethical guidelines for animal experiments and the safety guidelines for research. The experiments complied with the Basel Declaration, Convention on the Trade in Endangered Species of Wild Fauna and Flora and the IUCN Policy Statement on Research Involving Species at Risk of Extinction. All research performed conforms to the ethical guidelines by the International Council for Laboratory Animal Science (ICLAS).

### Statistical processing

The measurement data were expressed using the form of ξ ± σ, compared groups with each other using *t* test, and then again performed comparison one more time within groups using variance analysis. The relationship between variable and variable was also researched. All statistical processes were completed with software package whose type was SPSS11.0. If P < 0.05, it is considered that there had significant difference. If P < 0.01, it is considered that there is a highly significant difference.

## Results

The statistics of the specimens’ weights were showed in Table 1, which summarized the weights of rats. There was no obvious difference in the weights of rats between the sham group and the groups of balloon injury. Situation of intimal hyperplasia was showed in Table 2. Analysis showed that the ratio value of intimal thickness and media thickness increased significantly after balloon injury.

**Table 1:** Weights of rats in each group (ξ ± ΣΔ)

	**Sham groups**	**First day group**	**4th day group**	**7th day group**	**14th day group**	**28th day group**
Sample No.	8	8	8	8	8	8
Weight (g)	434.15 ± 10.4*	434.68 ± 17.2*	434.17 ± 11.3*	434.28 ± 18.38*	434.37 ± 15.1*	434.24 ± 13.7*

There was no significant difference in body weights of rats in each group (*P > 0.05).

**Table 2:** Changes of ratio of intimal thickness and media thickness (ξ ± ΣΔ)

	**Sham group**	**4th day group**	**7th day group**	**14th day group**	**28th day group**
Sample No.	8	8	8	8	8
Intimal thickness/media thickness	0.03 ± 0.01	0.19 ± 0.04*	0.35 ± 0.05*	0.66 ± 0.07*	0.91 ± 0.15*

On 4, 7, 14 and 28 days after balloon injury, ratios of intimal thickness and media thickness were higher compared with the sham group and the differences were significant (*P < 0.05).

**Figure 1:**
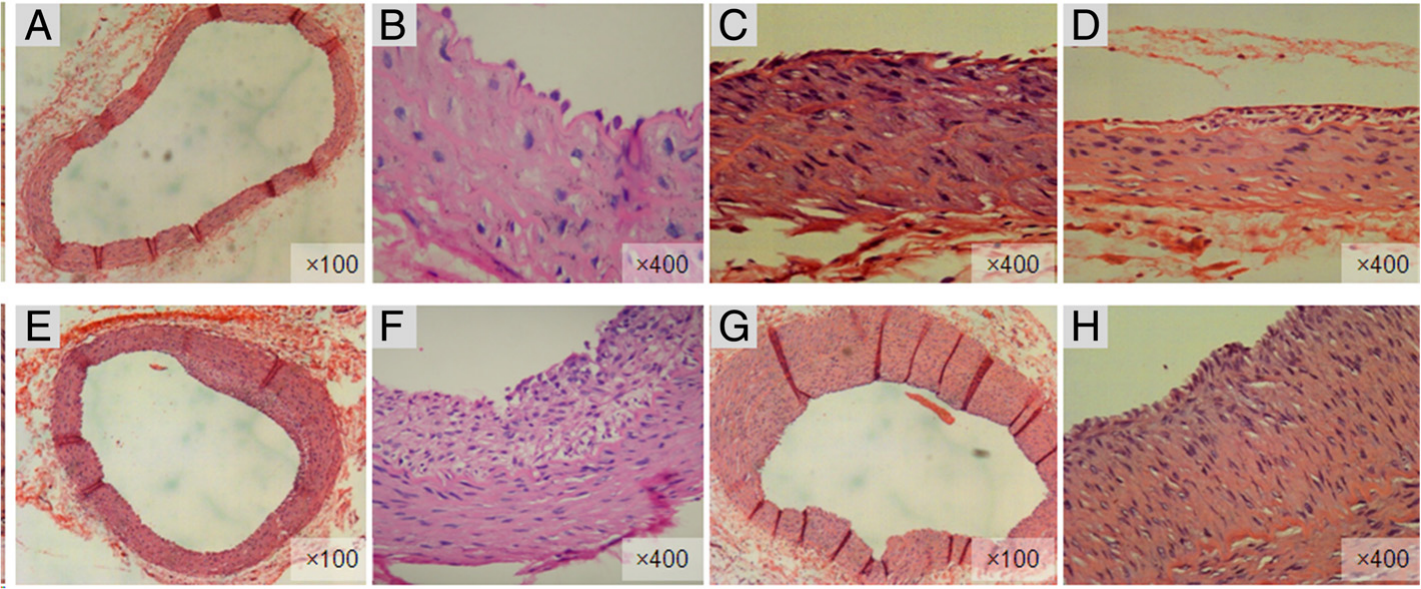
The results of H&E staining of pathology morphological examination of vascular in sham group and in the balloon injury group. **A**, **B** In the sham group, the intima was smooth, the internal elastic lamina was intact (H&E staining × 100), and endothelial cells were visible (H&E staining × 400); **(C)**: 4 days after balloon injury, elastic plate fractures were visible and medial smooth muscle cells migrated to the intima (H&E staining × 400); **(D)**: 7 days after balloon injury, a few of neointimals grew into the lumen (H&E staining × 400); **(E)**: 14 days after balloon injury, hyperplasia of intima was faster and the lumen began to narrow (H&E staining × 100); **(F)**: 14 days after balloon injury, hyperplasia of intima was faster and there were more vascular smooth muscle cells and extracellular matrix in neointimal (H&E staining × 400); **(G)**: 28 days after balloon injury, intimal hyperplasia was significant and lumen reduced significantly (H&E staining × 100); **(H)**: 28 days after balloon injury, there were many vascular smooth muscle cells and extracellular matrix in neointimal (H&E staining × 400).

Both H&E staining and elastic fiber staining were performed to all the animals. The results of HE staining of vascular pathology morphological examination were divided into sham group and balloon injury group and were demonstrated in Figure 1. As shown in Figure 1A and B, in the sham group, the intima was smooth, the internal elastic lamina was intact, and endothelial cells were visible. In the subgroup of 4 days after balloon injury, elastic plate fractures were visible and medial smooth muscle cells migrated to the intima (Figure 1C). In the subgroup of 7 days after balloon injury, a few of neointimals grew into the lumen (Figure 1D). In the subgroup of 14 days after balloon injury, hyperplasia of intima was faster and the lumen began to narrow (Figure 1E). Also, hyperplasia of intima was faster and there were more vascular smooth muscle cells and extracellular matrix in neointimal (Figure 1F). In the subgroup of 28 days after balloon injury, intimal hyperplasia was significant and lumen reduced significantly (Figure 1G), and there were many vascular smooth muscle cells and extracellular matrix in neointimal (Figure 1H).

**Figure 2:**
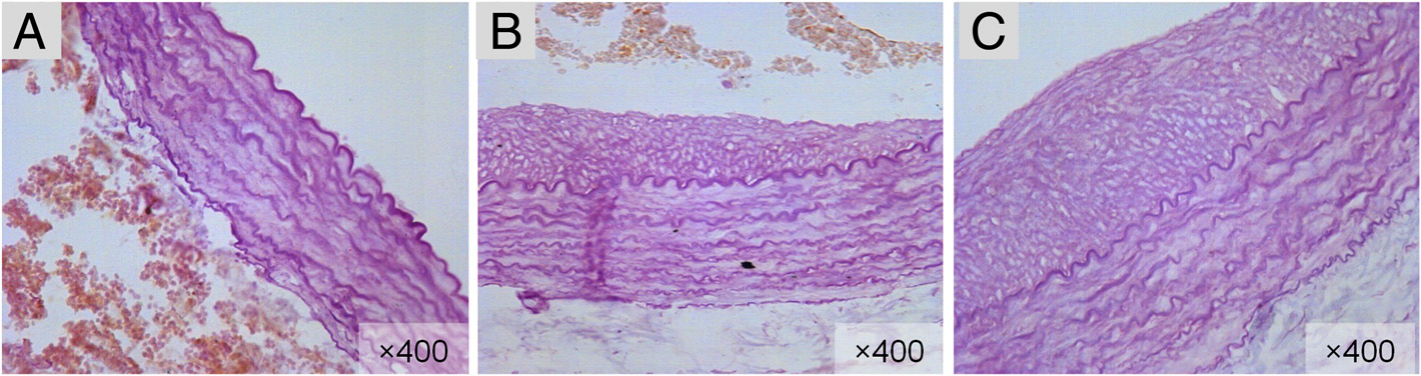
The Results of elastic fiber staining of pathology morphological examination of vascular in sham group and in the balloon injury group. **A**: Sham group, there was no intimal hyperplasia in sham group (elastic fiber staining × 400); **(B)**: 14 days after balloon injury, intimal hyperplasia was significant (elastic fiber staining × 400); **(C)**: 28 days after balloon injury, intimal hyperplasia was significant (elastic fiber staining × 400).

The results of Elastic Fiber staining were showed in Figure 2. In the sham group, there was no intimal hyperplasia in sham group (Figure 2A). In the subgroup of 14 days after balloon injury, intimal hyperplasia was significant (Figure 2B). In the subgroup of 28 days after balloon injury, intimal hyperplasia was significant (Figure 2C).

**Figure 3:**
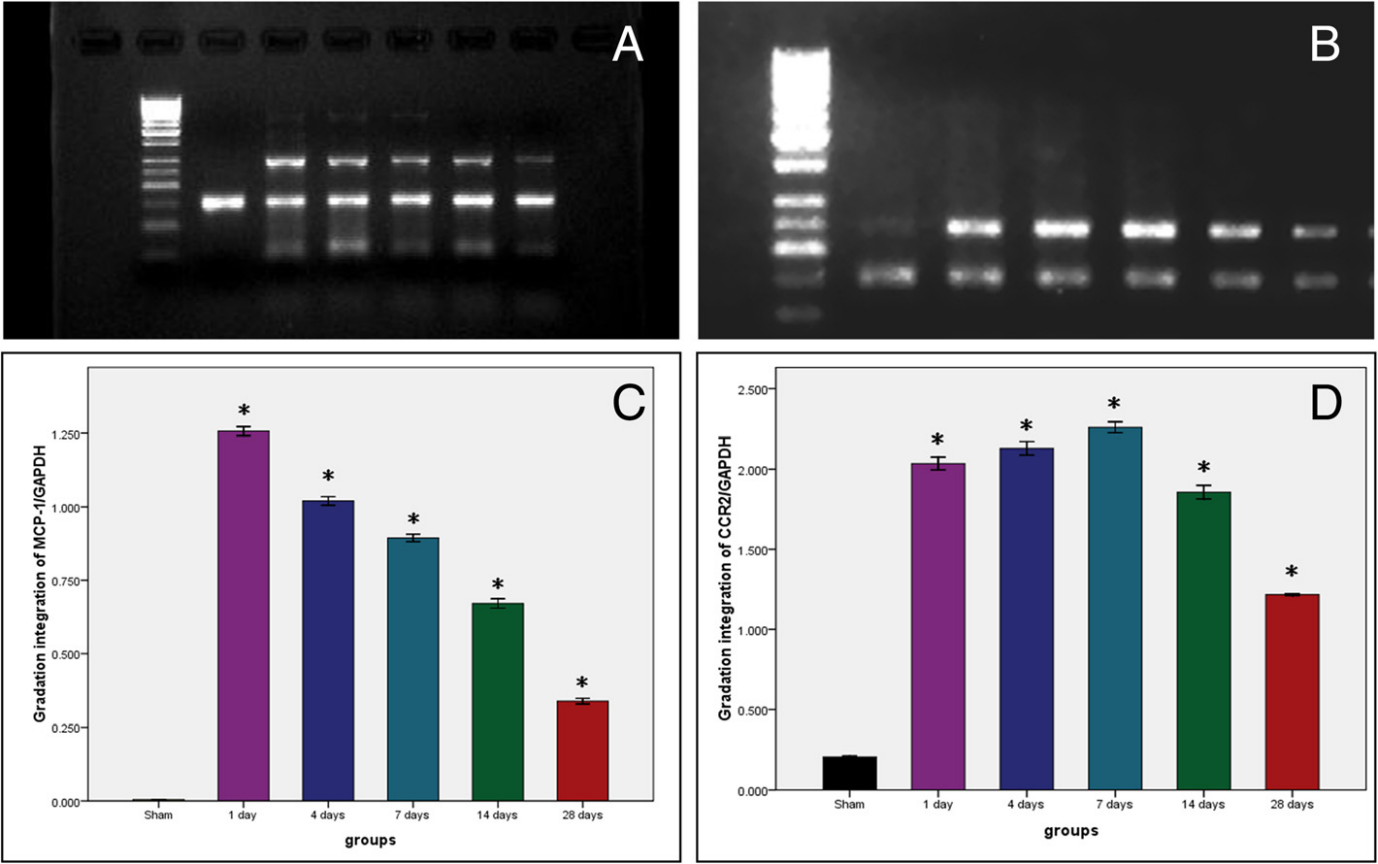
Results of PCR and identification of products. **A**: electrophoretogram of MCP-1 PCR amplification products at different time points. From left to right respectively were: M: 50 bp DNA Marker, 1: sham group, 2: first day group, 3: 4th day group, 4: 7th day group, 5: 14th day group, 6: 28th day group; **(B)**: trend diagram of gradation integration of MCP-1/GAPDH, from left to right respectively represented sham group, first day group, 4th day group, 7th day group, 14th day group and 28th day group; **(C)**: electrophoretogram of CCR2 PCR amplification products at different time points. From left to right respectively were: M: 50 bp DNA Marker, 1: sham group, 2: first day group, 3: 4th day group, 4: 7th day group, 5: 14th day group, 6: 28th day group; **(D)**: trend diagram of gradation integration of CCR2/GAPDH, from left to right respectively represented sham group, first day group, 4th day group, 7th day group, 14th day group and 28th day group.

The results of PCR and identification of product were showed in Figure 3. Corresponding to the electrophoretogram of MCP-1 PCR amplification products (Figure 3A), there was no expression of MCP-1 mRNA in sham group. The expressions of MCP-1 mRNA were detected in all balloon injury groups. As shown in Figure 3C, the expression of MCP-1 mRNA decreased with time. Compared with the sham group, expressions of MCP-1 mRNA increased in the balloon injury group (*P < 0.01, **P < 0.05). Corresponding to the results of electrophoretogram of CCR2 PCR amplification products, trace expressions of CCR2 mRNA could be detected in both sham group and balloon injury group (Figure 3B). Much larger amounts of expressions of CCR2 mRNA can be detected in the balloon injury groups. As shown in Figure 3D, the expressions of CCR2 mRNA gradually increased and maximized at the 7th day, and then began to decrease. By the 28th day, large amounts of expressions of CCR2 mRNA can still be detected. Compared with the sham group, expressions of MCP-1 mRNA increased in the balloon injury group (*P < 0.01, **P < 0.05).

**Figure 4:**
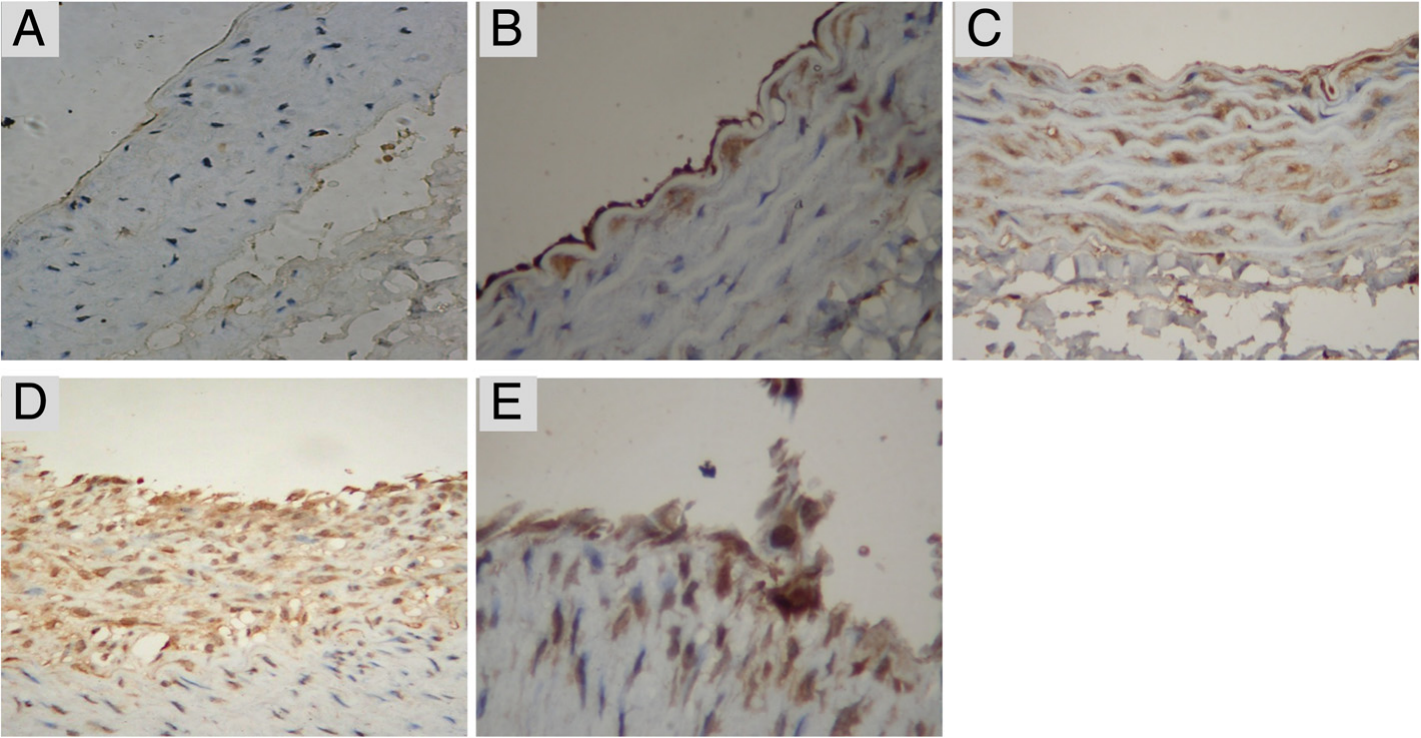
Detection results of immunohistochemistry of MCP-1 proteins in the vessel walls. **A**: negative expressions of MCP-1 proteins in sham group (Immunohistochemical SP method, DAB color, × 400); **(B)**: positive expressions of MCP-1 proteins in cytoplasm of vascular smooth muscle cells near the internal elastic plates after balloon injury for 4 days (Immunohistochemical SP method, DAB color, × 400); **(C)**: positive expressions of MCP-1 proteins in cytoplasm of smooth muscle cells in media after balloon injury for 7 days (Immunohistochemical SP method, DAB color, × 400); **(D)**: positive expressions of MCP-1 proteins in neointimal after balloon injury for 14 days (Immunohistochemical SP method, DAB color, × 400); **(E)**: positive expressions of MCP-1 proteins in neointimal after balloon injury for 28 days (Immunohistochemical SP method, DAB color, × 400).

The results of immunohistochemistry of MCP-1 proteins in the vessel walls were shown in Figure 4. There was no expression of MCP-1 proteins in sham group (Figure 4A). After balloon injury, expressions of MCP-1 proteins gradually increased and the expressions in cytoplasm of vascular smooth muscle cells near the internal elastic plates increased on the 4th day (Figure 4B). On the 7th day, large amounts of expressions of MCP-1 proteins could be detected in cytoplasm of smooth muscle cells in media (Figure 4D). On the 14th day, expressions in neointimal reached to the peak. on the 28th day, large amounts of expressions could be detected in neointimal.

**Figure 5:**
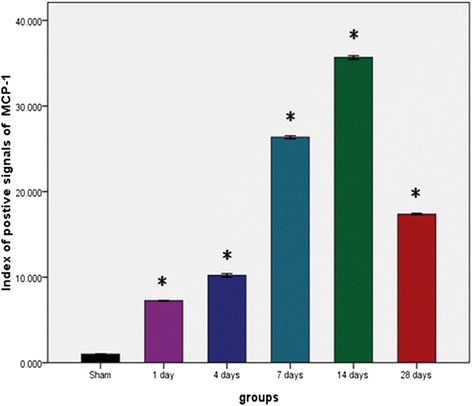
The index of positive signals of MCP-1 proteins by the semiquantitative method of immunohistochemistry in the arterial tissues of rats in six groups. We compared expression of MCP-1 with different experimental groups and compared with the sham group, (*P < 0.01).

Figure 5 shows the index of positive signals of MCP-1 proteins by the semiquantitative method of immunohistochemistry in the arterial tissues of rats in six groups. The expression of MCP-1 were compared with different experimental groups and compared with the sham group, (*P < 0.01). The plot shows an increasing trend from the sham group to the 14th day group, and then decreased for the 28th day group (*P < 0.05, **P < 0.01).

**Figure 6:**
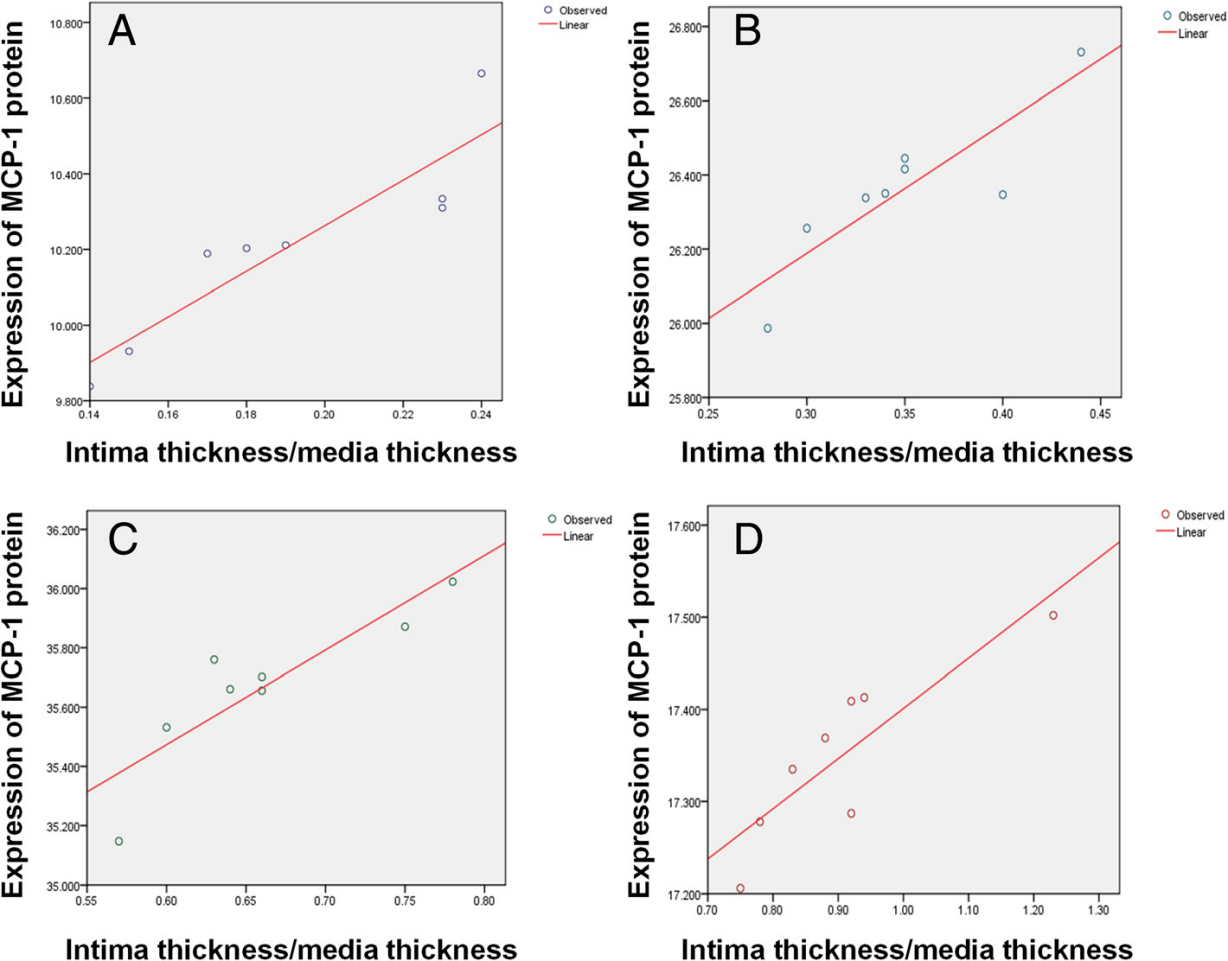
Correlation between expressions of MCP-1 proteins and intimal hyperplasia (the ratio of intimal thickness and media thickness) for 4, 7, 14 and 28 days. The related coefficients respectively were 0.831 (*P* = 0.002), 0.869 (*P* = 0.005), 0.880 (*P* = 0.004) and 0.864 (*P* = 0.006) as shown by images **A**, **B**, **C** and **D**.

The correlation of expressions of MCP-1 proteins and index of intimal hyperplasia (the ratio of intimal thickness and media thickness) were plotted and shown in Figure 6. The results of related analysis of Pearson showed that after balloon injury for 4, 7, 14 and 28 days, the related coefficients respectively were 0.831 (P = 0.002), 0.869 (P = 0.005), 0.880 (P = 0.004) and 0.864 (P = 0.006). The results showed that the expressions of MCP-1 proteins have an obvious positive correlation to the intimal hyperplasia after balloon injury.

## Discussions

PTCA that is recognized as an effective method for treating stenosis of coronary artery is widely used in clinic, but the high rate of restenosis has already been a thorny and difficult issue which is faced by physicians of cardiovascular intervention [14]. At present, the common method of making animal models of restenosis is that by simulating the process of PTCA which can result in the dilated injury of large and median arteries and pathological changes of intimal hyperplasia and stenosis [15].

In recent years, a large number of studies have shown that inflammatory response in the arterial wall plays an important role in the development of restenosis and chemoattractant and activation of monocyte which located in the arterial wall is an important pathological link in early reaction stage of vascular injury [16]. Chemokines are involved in the migration and activation of leukocytes especially of phagocytes and lymphocytes and play a vital role in inflammatory response [17,18]. According to the distribution and connection types of the two disulfide bonds of cysteine at both ends, they can be divided into CC, CXC, CX3C, C and other 4 sub families. MCP belong to the CC family of chemokine, which are divided into MCP-1, MCP-2, MCP-3, MCP-4, MCP-5, target cells in their respective effects are not the same, wherein MCP-1 is a major role in chemotactic factor of monocytes and macrophages [19,20]. MCP-1 can be secreted by a variety of cells, including osteoblasts, endothelial cells, smooth muscle cells, fibroblasts, monocytes, epidermal cells and some tumor cells. CCR2 is one of CC chemotactic cytokine receptors, mainly expresses in monocytes, kidney, heart, bone marrow, lung, liver and pancreas and other tissues [21]. CCR2 is the physiological receptor of MCP-1, after combining with MCP-1, it can induce activation of monocyte and takes the signal transduction pathway mediated by G protein coupled receptors.

It has been found that in this study 4 days after balloon injury, medial smooth muscle cells began to migrate to intima, on the 7th day, we could see moderate quantities of hyperplasia of vascular smooth muscle, on the 14th day to the 28th day, intima started to thicken obviously. Results of RT-PCR showed that there was no expression of MCP-1 mRNA in sham group and we could see trace expressions of CCR2 mRNA. After balloon injury, expressions of MCP-1 and CCR2 mRNA both significantly increased. The expressions of MCP-1 mRNA reached to the peak on the first day after balloon injury, following that, gradually decreased and it could still be detected on the 28th day. Expressions of CCR2 mRNA increased gradually after balloon injury and reached to the peak on the 7th day. After that, they decreased slowly and we could still detect large amounts of expressions of CCR2 mRNA on the 28th day. Results of immunohistochemistry showed that there was no expression of MCP-1 proteins in sham group. After balloon injury, expressions of MCP-1 proteins increased gradually. On the 4th day, expressions in cytoplasm of vascular smooth muscle cells near the internal elastic lamina increased. On the 7th day, we could detect a relatively large amount of expressions in the cytoplasm of smooth muscle cells in media. By the 14th day, we could detect significant expressions in the hyperplasia endometrium; on the 28th day, we could detect large amounts of expressions in the hyperplasia endometrium, but the expressions in smooth muscle cells in media decreased obviously. These results that expressions of MCP-1 and CCR2 increased in the repair process of vascular injury were consistent with foreign researches.

Currently, although the pathological mechanism of restenosis after PTCA has not yet been fully elucidated, excessive intimal hyperplasia caused by large proliferation of vascular smooth muscle cells and migration to intima which is induced by vascular endothelial injury is still widely considered to be the main pathological mechanism of restenosis after PTCA [22,23]. Our research demonstrates that the expressions of MCP-1 mRNA increased significantly after balloon injury and there were a lot of MCP-1 proteins expressed in the cytoplasm of smooth muscle cells and endometrial tissues, however we did not find the expression in sham group. Relevant analysis showed that the expressions of MCP-1 proteins had more significant positive correlation with intimal hyperplasia. This conclusion suggested that MCP-1 may participate in the migration and proliferation of VSMC and the occurrence and development of intimal hyperplasia. Porreca *et al.* found that MCP-1 is not only chemotactic activator but also the mitogen of vascular smooth muscle cells of rats, DNA analysis, cell count and cell division cycle analysis all have confirmed this conclusion [24]. Spinetti G *et al.* found that MCP-1 and CCR2 can promote the migration and proliferation of vascular smooth muscle cells of rats [25]. Various other researchers discovered that taking intervention measures, which can block the expressions of MCP-1, can inhibit the migration and proliferation of vascular smooth muscle cells after vascular injury, subsequently further reduce intimal hyperplasia. Zuoyun *et al.* found monoclonal antibody of MCP-1 can inhibit proliferation and migration of vascular smooth muscle cells, which mediate by angiotensin (AngII), through culture of cell *in vitro*. Then, Furukawa *et al.* found that antibody which can anti MCP-1can obviously inhibit the proliferation and migration of VSMC in carotid of rats after balloon injury [26]. In addition, Kim *et al.* reported that intimal hyperplasia in nude mice whose MCP-1 genes were knocked out after arterial injury significantly reduce compared with wild-type mice and confirmed that MCP-1 reduce the effect of intimal hyperplasia main through inhibiting the proliferation of vascular smooth muscle cells [27]. Now, 7ND is a variant of MCP-1 and its N-terminal miss 2 to 8 amino acids, it can combine with MCP-1, later on form to 7ND/MCP-1 heterodimer, further combine with CCR2, but lack physiological functions of MCP-1 and commonly is used to block the biological effects of MCP-1 [28]. Emiko *et al.* blocked the combining of MCP-1 and CCR2 by the method of injection 7ND to skeletal muscle, which can obviously inhibit the migration and proliferation of smooth muscle cells after arterial balloon injury caused by hyperlipidemia in rabbit [29]. These results of researches all strongly confirmed the pathway of signal transduction mediated by MCP-1 participated in the migration and proliferation of vascular smooth muscle cells and the occurrence and development of intimal hyperplasia from different angles. MCP-1 promote the migration and proliferation of vascular smooth muscle cells through which mechanism, now be not yet fully elucidated. Porreca *et al.* believed the main mechanisms is that: increasing the number and proportion of vascular smooth muscle cells in the S phase, meanwhile reducing the number of vascular smooth muscle cells in G0/G1 phase. By culturing vascular smooth muscle cells of human in vitro, Craig *et al.* found that MCP-1 lead to the proliferation of vascular smooth muscle cells mainly through increasing expressions of cyclin A and further effecting cell cycles [30]. The study also found that the expressions of CCR2 mRNA significantly increased after balloon injury, CCR2 is the major physiological receptor of MCP-1 and it plays an irreplaceable role in the process of MCP-1 play biological effects, these prompt CCR2 may also be involved in the proliferation of the intima after balloon injury.

So far, the mechanisms of MCP-1 and CCR2 in intimal hyperplasia has not yet been fully elucidated, conclusions that are obtained by interventions of MCP-1 and CCR2 are mostly based on animal models, but pathological environments of animal are not exactly same to human, so the exact mechanism of MCP-1 and CCR2 play a role in intimal hyperplasia and stenosis still need be further studied.

## Conclusion

In this study, the rat models of balloon injury were developed, the changes of intimal proliferation were observed with optical microscopy, and the expressions of MCP-1 and CCR2 at different times were examined with the methods of RT-PCR and immunohistochemistry. The expressions of MCP-1 and CCR2 in the arterial tissues were detected using reverse transcription polymerase chain reaction (RT-PCR) and analyzed by semi-quantitative method. The results showed that the dynamic expressions of MCP-1, MCP-1 proteins and CCR2 mRNA after balloon injury were shown to play an important role in intimal proliferation.
